# Nitric oxide activation facilitated by cooperative multimetallic electron transfer within an iron-functionalized polyoxovanadate–alkoxide cluster†
†Electronic supplementary information (ESI) available: ^1^H NMR and ^57^Fe Mössbauer spectra of **2-V_5_FeNO**, **3-V_5_FeNO^–^**, **4-V_5_FeNO^2–^**, **5-V_5_FeNO^+^**. Spectroscopic characterization for complex **6-V_5_FeNO^3–^** (^1^H NMR, solution IR). See DOI: 10.1039/c8sc00987b


**DOI:** 10.1039/c8sc00987b

**Published:** 2018-07-02

**Authors:** F. Li, R. L. Meyer, S. H. Carpenter, L. E. VanGelder, A. W. Nichols, C. W. Machan, M. L. Neidig, E. M. Matson

**Affiliations:** a Department of Chemistry , University of Rochester , Rochester , New York 14627 , USA . Email: matson@chem.rochester.edu; b Department of Chemistry , University of Virginia , Charlottesville , Virginia 22904-4319 , USA

## Abstract

A series of NO-bound, iron-functionalized polyoxovanadate–alkoxide (FePOV–alkoxide) clusters have been synthesized, providing insight into the role of multimetallic constructs in the coordination and activation of a substrate. Upon exposure of the heterometallic cluster to NO, the vanadium-oxide metalloligand is oxidized by a single electron, shuttling the reducing equivalent to the {FeNO} subunit to form a {FeNO}^7^ species. Four NO-bound clusters with electronic distributions ranging from [VV3VIV2]{FeNO}^7^ to [VIV5]{FeNO}^7^ have been synthesized, and characterized *via*^1^H NMR, infrared, and electronic absorption spectroscopies. The ability of the FePOV–alkoxide cluster to store reducing equivalents in the metalloligand for substrate coordination and activation highlights the ultility of the metal-oxide scaffold as a redox reservoir.

## Abstract 2

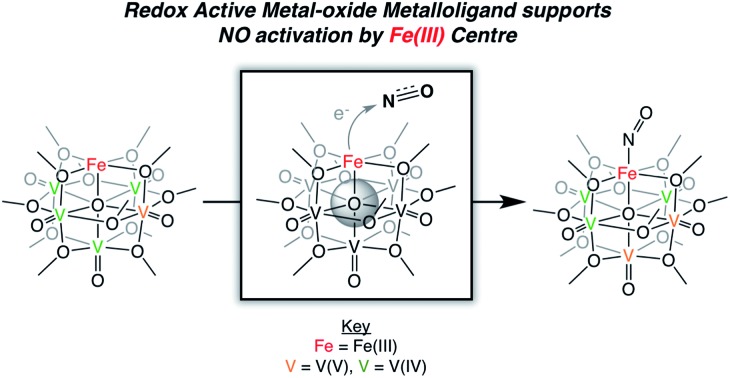
Cooperative multimetallic electron transfer to accommodate substrate binding.

## Introduction

**Fig. 1:**
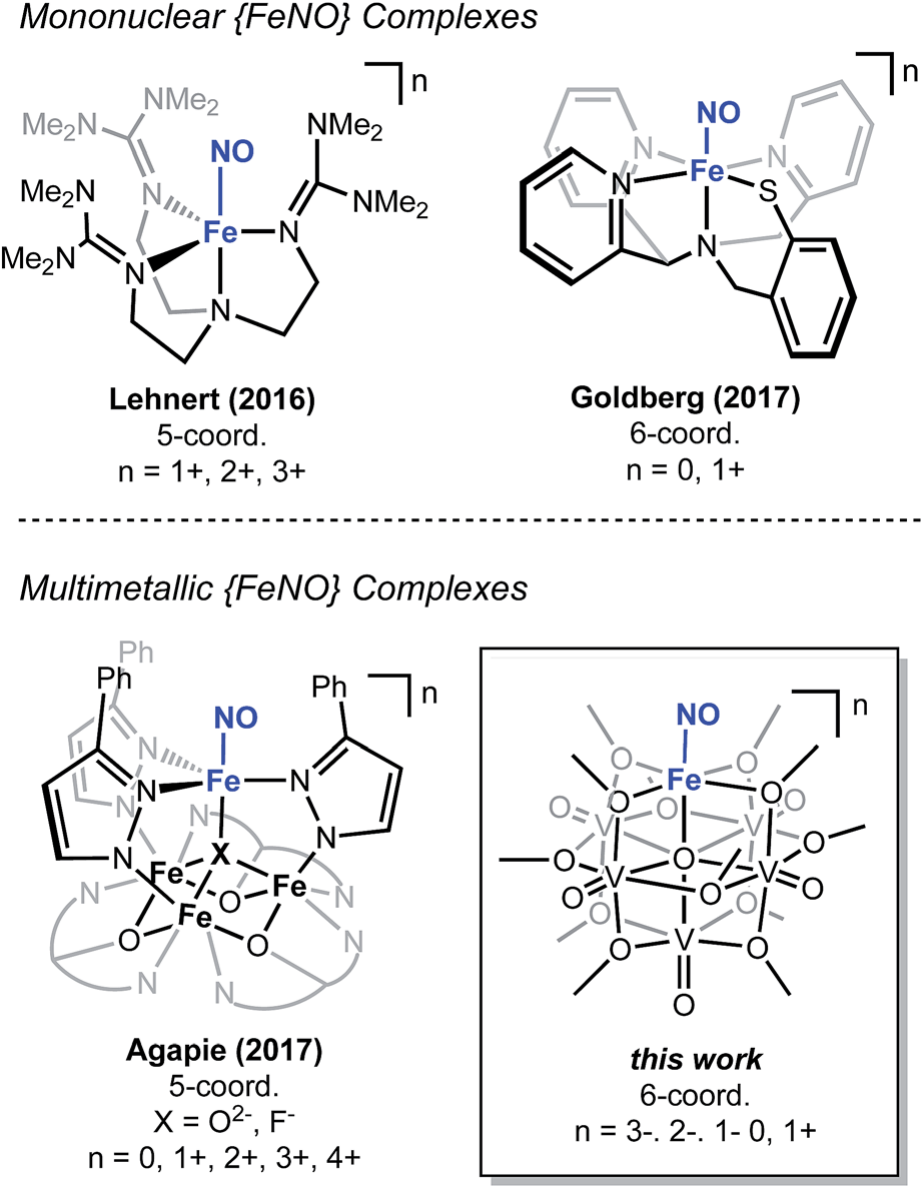
Select examples of non-heme high-spin 5- and 6-coordinate {FeNO} complexes.

The chemical reactivity of nitric oxide (NO) has captivated the field of bioinorganic chemistry, due to the participation of this small molecule in vasodilation, mammalian signalling, and immune defence processes.^1^ Given the established significance of this substrate in biological systems^2^,^3^ and the biogeochemical nitrogen cycle,^4^,^5^ the interaction of NO with metal centres, specifically iron, has been an area of intense research. Indeed, the prevalence of heme- and non-heme-containing metalloenzymes in NO reductases has driven interest in understanding the electronic structure of the {FeNO} subunit in metalloproteins and model complexes.^6^–^8^ A combination of spectroscopic, crystallographic, and theoretical methods have shown that substrate binding and reduction are key steps in NO activation.^2^,^3^,^9^–^13^ However, despite reports describing the behaviour of NO with ferric and ferrous heme complexes, the chemistry of this substrate with non-heme derivatives remains underdeveloped (Fig. 1). Toward a more complete understanding of the redox chemistry involved during NO activation, the synthesis, characterization, and reactivity of non-heme models, capable of supporting variable oxidation states of the {FeNO} subunit, are of interest.

The activation of NO requires the simultaneous transfer of multiple electrons and protons to the substrate. In nature, similar chemical transformations of gaseous substrates (*e.g.* H_2_, N_2_, CO_2_) are often mediated by metalloenzymes that feature multiple, closely associated metal centres within the active site.^14^–^20^ Cooperative, multimetallic reactivity has also been noted in heterogeneous systems, where reducible metal-oxide supports have been demonstrated to enhance the activity of metal-catalysts for the activation of small molecules.^21^–^23^ In an effort to design homogeneous complexes that mimic the activity of these reactive multimetallic assemblies, our group^24^,^25^ and others^26^–^31^ have recently reported heterometallic complexes that possess varying degrees of multimetallic electronic communication. Extensive structural and spectroscopic characterization of these clusters has illustrated the participation of the assembly in molecular redox tuning and the storage of electron density. While these studies have expanded our understanding of the static electronic properties of well-defined multinuclear systems, comparatively less is known about the role of electron transfer across a multimetallic assembly during substrate activation.

Recently, our research group has reported the synthesis and characterization of a family of iron-functionalized polyoxovanadate–alkoxide (FePOV–alkoxide) complexes (Fig. 1).^24^,^25^,^32^ Using a suite of analytical techniques, we have demonstrated that the metal-oxide metalloligand functions as a redox reservoir for the ferric centre, storing reducing equivalents across the vanadyl ions in a delocalized cloud of electron density. The established spectroscopic handles, unique to this heterometallic framework, provide the opportunity for *in situ* analysis of the oxidation state of the metal-oxide scaffold. Therefore, our FePOV–alkoxide complexes are ideal for the investigation of electron transfer within heterometallic assemblies during substrate activation. Given the well-described electronic structures of the {FeNO} subunit, and the large degree of substrate sensitivity to the electronic environment of the bound iron centre,^33^ we selected NO as an ideal substrate to investigate the reactivity of FePOV–alkoxide clusters. Herein, we report our results that showcase the ability of the POV–alkoxide scaffold to shuttle stored electron density to NO through the ferric centre. This work highlights the role of electron transfer within a multimetallic construct during chemical transformations and substrate activation.

## Results and discussion

### Reactivity of NO(g) with an FePOV–alkoxide cluster

**Scheme 1:**
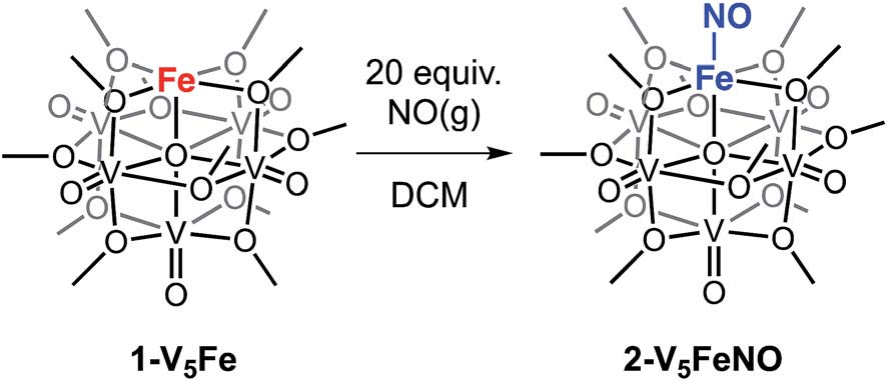
Reactivity of **1-V_5_Fe** with NO(g).

**Fig. 2:**
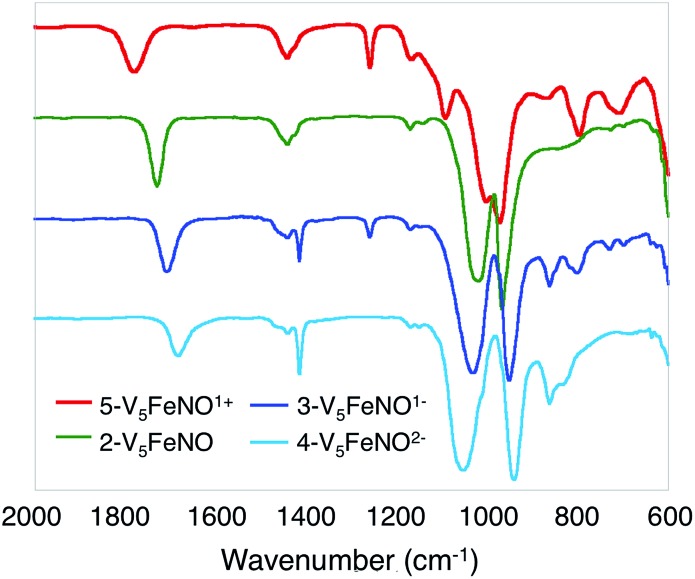
Infrared spectra of complexes **2-V_5_FeNO**, **3-V_5_FeNO^–^**, **4-V_5_FeNO^2–^** and **5-V_5_FeNO^+^**.

To investigate the reactivity of NO with high-spin ferric centres embedded within a multimetallic cluster, NO(g) was added to a solution of the neutral, FePOV–alkoxide cluster, [VIV4V^V^O_6_(OCH_3_)_12_Fe^III^] (**1-V_5_Fe**) (Scheme 1). A gradual colour change from dark-green to brown was observed. Analysis of the reaction mixture *via*^1^H NMR spectroscopy revealed formation of a new product, with three paramagnetically shifted and broadened resonances located at 13.86, 20.12, and 127.44 ppm (Fig. S1†). Formation of the desired NO adduct of the FePOV–alkoxide cluster, [V_5_O_6_(OCH_3_)_12_{FeNO}] (**2-V_5_FeNO**), was confirmed by infrared spectroscopy. A new band corresponding to *v*(NO) was located at 1732 cm^–1^ (Fig. 2, Table 1), substantially shifted from that of free NO (1900 cm^–1^).^34^,^35^ Due to the coordinative saturation of the vanadyl ions of the FePOV–alkoxide cluster and the shift in *v*(NO) consistent with formation of an iron–nitrosyl subunit,^32^,^36^–^38^ we concluded that NO most likely binds to the cluster through the vacant site of the 5-coordinate, square pyramidal Fe(iii) centre of the multinuclear assembly (Scheme 1).

**Table 1:** Comparison of spectroscopic parameters for **2-V_5_FeNO**, **3-V_5_FeNO^–^**, **4-V_5_FeNO^2–^**, **5-V_5_FeNO^+^** and **6-V_5_FeNO^3–^** to selected {FeNO} complexes

Compound	{FeNO}^*n*^	CN^*a*^	Fe spin state	*v*(NO) (cm^–1^)	*v*(O_b_-CH_3_) (cm^–1^)	*ν*(V=O_t_) (cm^–1^)	*δ* (mm s^–1^)^*a*^	Δ*E*_q_ (mm s^–1^)^*a*^	Ref.
**5-V_5_FeNO^+^**	7	6		1780	1003	974	—^*b*^	—^*b*^	This work
**2-V_5_FeNO**	7	6		1732	1022	968	0.63^*c*^	0.88^*c*^	This work
**3-V_5_FeNO^–^**	7	6		1709	1032	951	0.59^*c*^	0.69^*c*^	This work
**4-V_5_FeNO^2–^**	7	6		1683	1055	935	0.56^*c*^	0.49^*c*^	This work
[(TMG_3_tren){FeNO}]^3+^	6	5	h.s.	1879	—	—	0.06^*d*^	0.48^*d*^	19
[(TMG_3_tren){FeNO}]^2+^	7	5	h.s.	1750	—	—	0.48^*d*^	1.42^*d*^	19
[(TMG_3_tren){FeNO}]^1+^	8	5	h.s.	1618	—	—	0.84^*d*^	2.78^*d*^	19
[(^Me3^TACN)Fe(NO)(N_3_)_2_]	7	6	l.s.	1690, 1712	—	—	0.62^*c*^	1.28^*c*^	25
*cis*-[(cyclam)Fe(NO)I]I	7	6	l.s.	1726	—	—	0.64^*c*^	1.78^*c*^	25
[LFe_3_F(PhPz)_3_Fe(NO)]^3+^^*e*^	7	5	h.s.	1842	—	—	0.62^*f*^	1.51^*f*^	56
[LFe_3_F(PhPz)_3_Fe(NO)]^2+^^*e*^	7	5	h.s.	1823	—	—	0.62^*f*^	1.39^*f*^	56
[LFe_3_F(PhPz)_3_Fe(NO)]^1+^^*e*^	7	5	h.s	1799	—	—	0.63^*f*^	1.67^*f*^	56
[LFe_3_F(PhPz)_3_Fe(NO)]^*e*^	8	5	h.s.	1680	—	—	0.95^*f*^	1.63^*f*^	56

^*a*^Coordination number.

^*b*^Complex readily decomposes. Isolation of this molecule for analysis by Mössbauer spectroscopy was not possible.

^*c*^Mössbauer spectrum measured at 80 K.

^*d*^Mössbauer spectrum measured at 4.2 K in frozen 1 : 1 propionitrile : butyronitrile solution.

^*e*^L = 1,3,5-tris(2-di(2′-dipyridyl)hydroxymethylphenyl)benzene.

^*f*^Mössbauer spectrum measured at 80 K in solid sample (polycrystalline material mixed with boron nitride). Parameter listed is for the apical iron centre bound to NO.

**Fig. 3:**
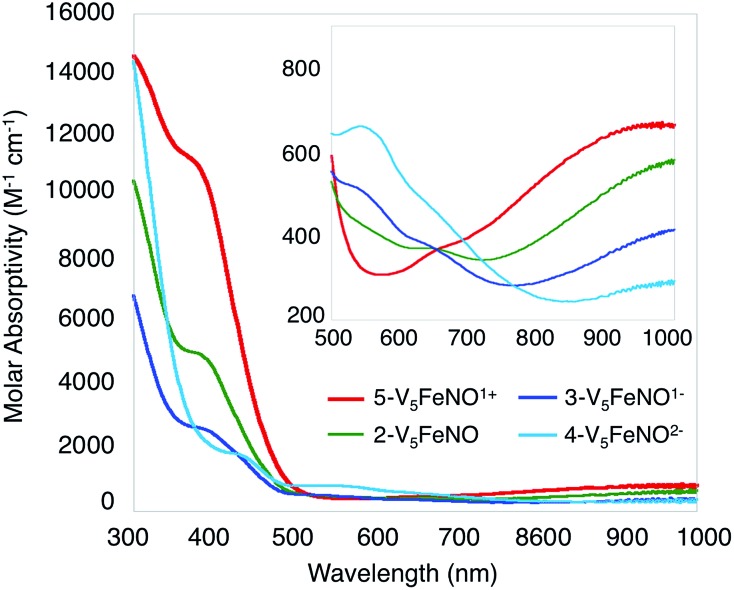
Electronic absorption spectrum of complexes **2–5** collected in CH_3_CN at 21 °C (the inset shows the low-energy region of the spectrum to more clearly illustrate IVCT bands).

Given the propensity of NO to disrupt the molecular structure of self-assembled, cluster complexes,^39^,^40^ retention of the polynuclear Lindqvist core following exposure of **1-V_5_Fe** to NO was evaluated by IR and electronic absorption spectroscopies. Two characteristic bands of the Lindqvist core were observed in the IR spectrum of **2-V_5_FeNO**, corresponding to *v*(V=O_t_) and *v*(O_b_–CH_3_) at 968 and 1022 cm^–1^, respectively (O_t_ = terminal vanadyl oxygen atoms, O_b_ = bridging oxygen atoms of methoxide ligands) (Fig. 2, Table 1). Additionally, the electronic absorption spectrum of **2-V_5_FeNO** reveals two intervalence charge-transfer (IVCT) bands, located at 382 nm (*ε* = 5.06 × 10^3^ M^–1^ cm^–1^) and 982 nm (*ε* = 5.80 × 10^2^ M^–1^ cm^–1^) (Fig. 3). These features correspond to *d*_*xy*_(V^IV^) → *d*_*x*^2^–*y*^2^_(V^V^) and *d*_*xy*_(V^IV^) → *d*_*xy*_(V^V^) electronic transitions, respectively. Previous reports have established that these absorptions are characteristic of mixed-valent, POV–alkoxide clusters, thus demonstrating retention of the Robin and Day Class II delocalization of electron density across the cluster core upon coordination of NO.^25^,^41^ Collectively, IR and electronic absorption spectroscopy confirm retention of the multimetallic assembly following substrate activation.^24^,^25^,^41^,^42^

The reactivity of complex **1-V_5_Fe** and NO is surprising, as metalloproteins and model complexes containing high-spin ferric centres have been reported to have a lower affinity for this substrate, in comparison to their ferrous congeners.^43^,^44^ This unusual binding of substrate at an Fe^III^ ion prompted the investigation into the participation of the metal-oxide metalloligand in substrate activation. To establish the role of multimetallic, cooperative reactivity, a five coordinate ferric complex, Fe^III^(salen)Cl (salen = *N*,*N*′-bis(salicylidene)ethylenediamine) was reacted with NO(g).^45^ This monometallic complex was selected as a suitable model for the FePOV–alkoxide clusters, due to their similar iron coordination environments (pseudo-square pyramidal) and the redox non-innocence of the respective organic and inorganic ligands. However, exposure of Fe^III^(salen)Cl to 20 equiv. of NO(g) yields no reaction. The disparity in the reactivity of the two, 5-coordinate, high-spin ferric complexes (Fe^III^(salen)Cl and **1-V_5_Fe**) indicates that the POV–alkoxide scaffold is directly involved in NO activation, most likely through the efficient transfer of electron density to the iron centre (*vide infra*).

### Analysis of reduced and oxidized derivatives of **2-V_5_FeNO**

**Fig. 4:**
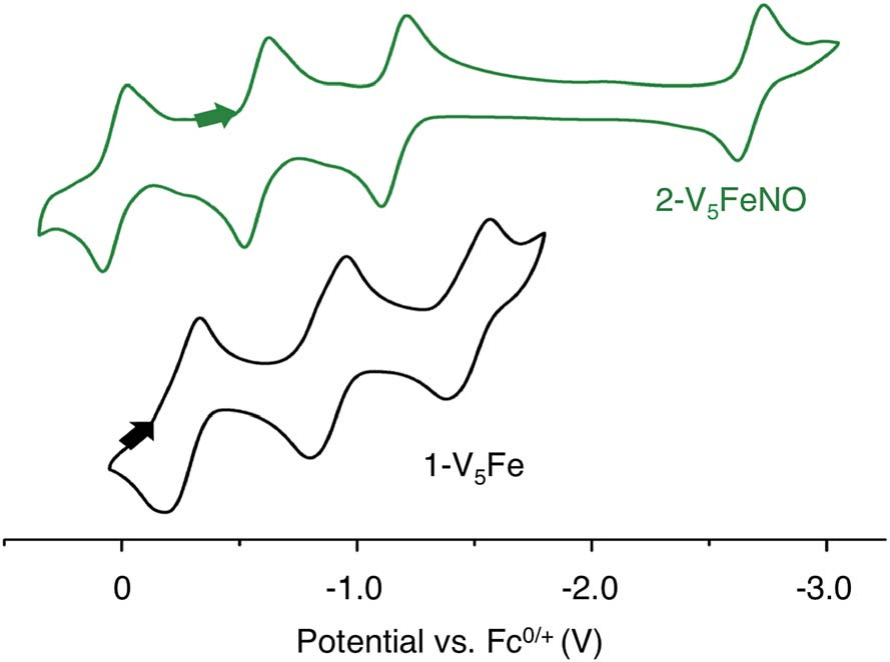
Cyclic Voltammogram of complexes **1-V_5_Fe** and **2-V_5_FeNO** collected in THF with 0.4 M [^*n*^Bu_4_N][PF_6_] as the supporting electrolyte (*v* = 0.1 V s^–1^).

Elucidation of the oxidation state distribution of metal ions in complex **2-V_5_FeNO** is necessary to define the role of the vanadium-oxide metalloligand ligand during NO activation. Previously, our group^25^ and others^41^,^42^,^46^ have demonstrated that the isolation of the reduced and oxidized derivatives of multimetallic complexes aids in the characterization of the charge distribution of metal ions that compose the cluster core. To assess the accessibility of a redox series of the NO-functionalized FePOV–alkoxide ({FeNO}POV–alkoxide) clusters, the electrochemical profile of **2-V_5_FeNO** was explored *via* cyclic voltammetry (CV). A voltammogram possessing four redox events located at *E*_1/2_ = +0.03, –0.58, –1.16, and –2.68 V *vs.* Fc/Fc^+^ was observed (Fig. 4). The reversibility of these events was established by varying the scan rate from 20 to 500 mV s^–1^ and fitting the data to the Randles–Sevcik equation (Fig. S2†). Linearity of each fully-reversible redox event was confirmed by plotting the current density (*j*_p_ reported in A cm^–2^) *versus* the square root of the scan rate (*v*^1/2^ reported in V s^–1^).^25^,^47^ Comparison of the CV of complex **2-V_5_FeNO** to that of the starting material, **1-V_5_Fe**, illustrates the drastic changes in the redox properties of the cluster upon coordination of NO, suggesting substrate coordination has a direct effect on the electronic structure of the multimetallic assembly.

**Scheme 2:**
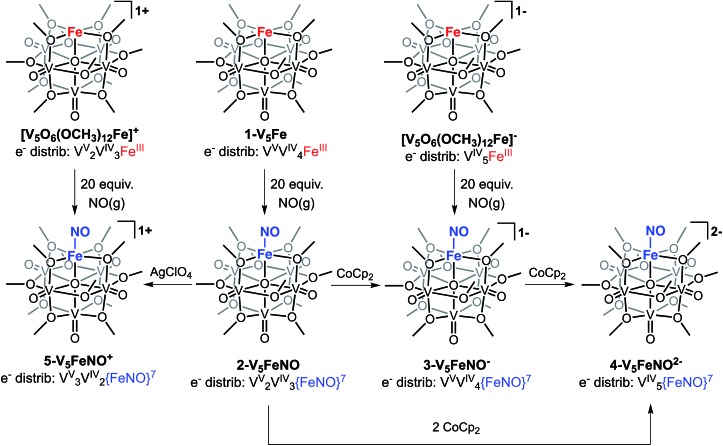
Synthesis of complexes **2-V_5_FeNO**, **3-V_5_FeNO^–^**, **4-V_5_FeNO^2–^**, and **5-V_5_FeNO^+^**.

The first three redox events in the CV of complex **2-V_5_FeNO**, observed at *E*_1/2_ = +0.03, –0.58, and –1.16 V, possess peak-to peak separations (Δ*E*_1/2_) of approximately 0.60 V. Similar values of Δ*E*_1/2_ have been observed in the case of FePOV–alkoxide clusters^25^ and their homometallic, hexavanadate congeners,^41^,^42^ and are associated with redox events localized to the vanadyl ions. In the case of complex **2-V_5_FeNO**, the Δ*E*_1/2_ values for these redox events are similar to those reported for the parent complex, **1-V_5_Fe**, resulting in a similar range of comproportionation constants (*K*_c_) between the two series of FePOV–alkoxide clusters (*K*_c_ = 8.8 × 10^9^ to 2.7 × 10^10^; RT ln(*K*_c_) = *nF* [Δ*E*01/2]).^48^ To unambiguously corroborate the hypothesis that the evenly spaced redox events correspond to vanadium-based electrochemical processes, the chemically reduced and oxidized derivatives of **2-V_5_FeNO** were isolated. Exposure of **2-V_5_FeNO** to stoichiometric equivalents of cobaltocene afforded access to the one- and two-electron reduced species, namely **3-V_5_FeNO^–^** and **4-V_5_FeNO^2–^** (Scheme 2). Oxidation of **2-V_5_FeNO** with AgClO_4_ at –40 °C led to the formation of **5-V_5_FeNO^+^**. The NO adducts (**3-V_5_FeNO^–^** and **5-V_5_FeNO^+^)** could be independently synthesized by reaction of NO(g) with the appropriate FePOV–alkoxide precursor ([V_5_O_6_(OCH_3_)_12_Fe]^–^ and [V_5_O_6_(OCH_3_)_12_Fe]^+^, respectively) (Scheme 2). In all cases, the products were analysed by ^1^H NMR spectroscopy (Fig. S3–S5†).

Upon sequential reduction from **5-V_5_FeNO^+^** to **4-V_5_FeNO^2–^**, the IR spectra reveal that *v*(V=O_t_) decreases from 974 to 935 cm^–1^, with corresponding increases in the values of *v*(O_b_–CH_3_) (1003 to 1055 cm^–1^) (Fig. 2, Table 1). Similar changes in the *v*(V=O_t_) and *v*(O_b_–CH_3_) stretching frequencies have been reported for the redox series of homo- and heterometallic POV–alkoxide complexes.^25^,^41^,^42^ Furthermore, with each electron added to the system, *v*(NO) shifts ∼30 cm^–1^ toward lower energies (Fig. 2, Table 1). This small change in stretching frequency of the nitrosyl ligand is inconsistent with the direct reduction of the iron–nitrosyl moiety, as this change in oxidation state of the {FeNO} subunit is typically associated with changes in *v*(NO) of ∼100 cm^–1^.^37^,^38^,^49^ Instead, the decrease in stretching frequency of *v*(NO) compares favourably to changes observed in the {FeNO}^6/7^ dithiolene systems.^50^,^51^ The redox active dithiolene ligands of these complexes are able to store equivalents of electron density upon sequential reduction from the mono-cationic to the mono-anionic species, [Fe(NO)(S_2_C_2_R_2_)_2_]^*n*^ (R = *p*-tolyl; *n* = +1, 0, –1). As a result of ligand participation in the reduction events of the complex, retention of the {FeNO}^6^ oxidation state was observed throughout the series. IR spectroscopy of the {FeNO} dithiolate complexes revealed small shifts in *v*(NO) (∼35 cm^–1^/e^–^), consistent with the changes in *v*(NO) observed in the spectra of the complexes **2–5**. These small, yet distinct changes are a consequence of the dependence of the electronic structure of the {FeNO} moiety on the oxidation state of the redox-active ligand.

Likewise, electronic storage across a metal-oxide base has been observed previously, in the case of [LFe_3_(PhPz)_3_EFeNO]^*n*+^ (L = 1,3,5-triarylbenzene ligand motif, E = O, F).^56^,^57^ Agapie and coworkers demonstrate through spectroscopic and crystallographic investigations that changes in oxidation state of the tetra-iron cluster, [LFe_3_(PhPz)_3_OFeNO]^1+^, occur exclusively across the distal iron centres. Mössbauer parameters of complexes [LFe_3_(PhPz)_3_OFeNO]^*n*+^ (*n* = 1, 2, 3) reveal little change in the isomer shift of the apical iron centre upon oxidation of the cluster core, indicating the retention of the {FeNO}^7^ state for the iron–nitrosyl adduct across the redox series. Furthermore, nominal changes in *v*(NO) (∼30 cm^–1^/e^–^) corroborate that no change in the oxidation state of the {FeNO}^7^ subunit occurs during cluster oxidation. Collectively, these results establish the role of the tri-iron base as a redox-reservoir for the apical iron centre. The POV–alkoxide scaffold acts in analogy to the iron-oxide base of [LFe_3_(PhPz)_3_OFeNO]^1+^ during changes in oxidation state of the cluster, as established *via* infrared analysis.

The electronic absorption spectra of complexes **2–5** provide additional insight into the electronic structure of the heterometallic Lindqvist cluster. Absorptions diagnostic of IVCT events between V(iv) and V(v) centres are observed in complexes **2-V_5_FeNO**, **3-V_5_FeNO^–^**, and **5-V_5_FeNO^+^**, suggesting the presence of a mixed-valent POV–alkoxide core.^25^,^41^,^42^ In previous work, similar features have been considered tell-tale signs of extensive delocalization of electron density across the POV–alkoxide assembly,^25^,^41^,^42^,^46^,^52^ In contrast, the electronic absorption spectrum of **4-V_5_FeNO^2–^** has no IVCT absorptions. Instead, the spectrum contains a weak feature at 542 nm (*ε* = 661 M^–1^ cm^–1^), which has been previously assigned to a forbidden *d*_*xy*_(V^IV^) → *d*_*x*^2^–*y*^2^_(V^IV^) excitation of an isovalent POV–alkoxide cluster.^25^ As such, the charge distribution for complex **4-V_5_FeNO^2–^** can be described as [VIV5O_6_(OCH_3_)_12_{FeNO}]^2–^. Taking into consideration that the sequential one-electron oxidations occur across the POV–alkoxide framework, the oxidation state distributions of the vanadium ions in the remaining {FeNO}POV–alkoxide clusters can be assigned as [V^V^VIV4O_6_(OCH_3_)_12_{FeNO}]^–^ (**3-V_5_FeNO^–^**), [VV2VIV3O_6_(OCH_3_)_12_{FeNO}] (**2-V_5_FeNO**), and [VV3VIV2O_6_(OCH_3_)_12_{FeNO}]^+^ (**5-V_5_FeNO^+^**) (Scheme 2). Thus, following exposure to NO, the POV–alkoxide core is oxidized by one electron, with respect to the parent cluster (Scheme 2).

### Electronic structure of the {FeNO} subunit

Following identification of the oxidation states of the vanadium ions within the POV–alkoxide assembly, charge balance reveals, in all cases, an overall 2^+^ charge on the {FeNO} subunit for complexes **2–5**. The required charge is satisfied by multiple resonance structures, namely [Fe^II^NO˙]^2+^ and [Fe^III^NO^–^]^2+^. Due to the ambiguity in the explicit oxidation state of the iron centre, the electronic structures of the iron–nitrosyl subunits of the FePOV–alkoxide complexes are best described as {FeNO}^7^ in the Enemark–Feltham notation.^53^ The *v*(NO) observed in the IR spectra of clusters **2–5** are consistent with this assignment, with values that resemble previously reported 6-coordinate {FeNO}^7^ complexes (Table 1).^11^

**Fig. 5:**
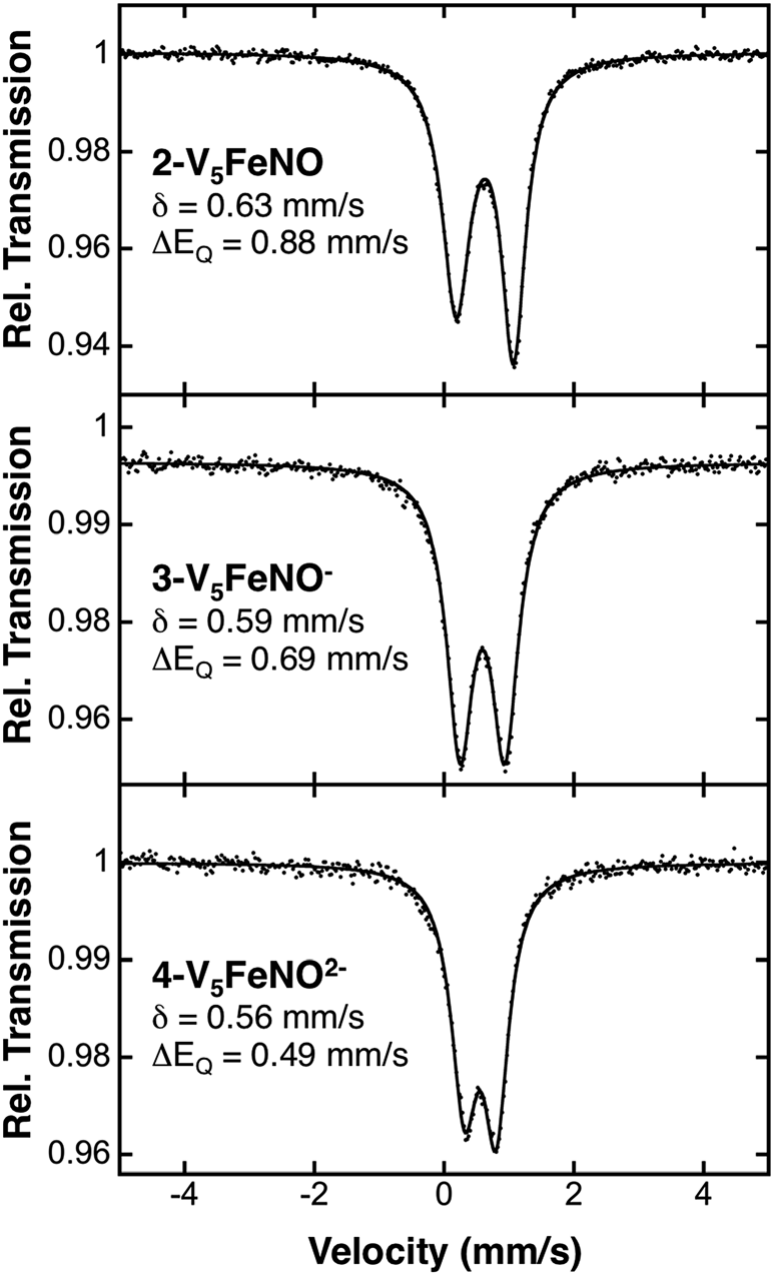
80 K Mössbauer spectra of complexes **2-V_5_FeNO**, **3-V_5_FeNO^–^**, and **4-V_5_FeNO^2–^**. For each spectrum, the data (dots) and fit (solid line) are given. The Mössbauer fit parameters for each spectrum are depicted with spectra, and also summarized in Table 1.

To more rigorously define the electronic structure of the iron–nitrosyl moiety, zero-field Mössbauer spectroscopy was performed on solid samples of complexes **2–4** (collected at 80 K) (Fig. 5). The broad, slightly asymmetric, quadrupole doublets resemble values reported previously for monodisperse FePOV–alkoxide clusters, indicating a single electronic environment for the iron centre.^25^ It is worth noting that the isomer shifts obtained from the Mössbauer spectra of the {FeNO}POV–alkoxide clusters resemble previously reported parameters for {FeNO}^7^ complexes (Table 1). The observed decrease in quadrupole splitting of the {FeNO}POV–alkoxide species as compared to their monometallic, 6-coordinate, {FeNO}^7^ congeners is likely reflective of the unique structure of the multimetallic cluster complexes.

Electron paramagnetic resonance (EPR) spectroscopy was also performed on complex **3-V_5_FeNO^–^** in order to obtain further insight into the spin state of the {FeNO}^7^ unit of these complexes. In addition to a broad *S* = 1/2 signal attributed to the POV–alkoxide scaffold,^54^ significant signal intensity in the *g* ∼ 4 region is present, consistent with the presence of a high-spin *S* = 3/2 {FeNO}^7^ unit (Fig. S6†).

The formation of the {FeNO}^7^ subunit upon exposure of the FePOV–alkoxide clusters to NO(g) is remarkable, given addition of a nitrosyl ligand to a ferric centre would be expected to result in the formation of an {FeNO}^6^ subunit. Generation of the {FeNO}^7^ moiety is consistent with either a metal-centred or substrate-based reduction upon coordination of NO to the multimetallic species. The reduction of the iron nitrosyl moiety occurs simultaneously with the observed oxidation of the POV–alkoxide scaffold, thus we can conclude that the metal-oxide metalloligand transfers electron density to the iron centre to facilitate activation of the substrate. This observed electron transfer between metal-oxide scaffold and iron constitutes a rare example of cooperative multimetallic reactivity for substrate activation. Similar flexibility in site-differentiated multimetallic clusters has recently been reported by Agapie and coworkers.^55^ This report demonstrates the utility of electron transfer from a mixed-valent iron oxide base to a site-differentiated ferric ion upon coordination of CO, resulting in the formation of [LFe_3_O(PhIm)Fe(CO)_*n*_] (L = 1,3,5-triarylbenzene ligand motif; PhIm = 1-phenyl imidazole) complexes. In both examples, electron transfer clarifies that the metalloligand plays an important part in substrate activation, providing electron density to a ferric centre to facilitate substrate coordination and reduction.

### Attempts to access the fully reduced {FeNO}POV–alkoxide

Our attention turned toward identification of the fully reduced species, generated by the one-electron reduction of complex **4-V_5_FeNO^2–^**. Interest in characterizing the product of this reaction stems from the surprising peak-to-peak separation associated with the redox event centred at *E*_1/2_ = –2.68 V *vs.* Fc/Fc^+^ (Δ*E*_1/2_ = 1.52 V). Similar separations of redox events in heterometal-functionalized polyoxometalates have been attributed to changes in oxidation state of the distinct metal centre incorporated within the cluster.^58^,^59^ Consistent with these observations, the reducing nature of this electrochemical process suggests the reduction event is localized to the site-differentiated, iron–nitrosyl moiety. Accordingly, we hypothesized that reduction of the {FeNO}^7^ subunit would give rise to the formation of a rare, non-heme {FeNO}^8^ species.^36^–^38^,^60^–^63^

**Scheme 3:**
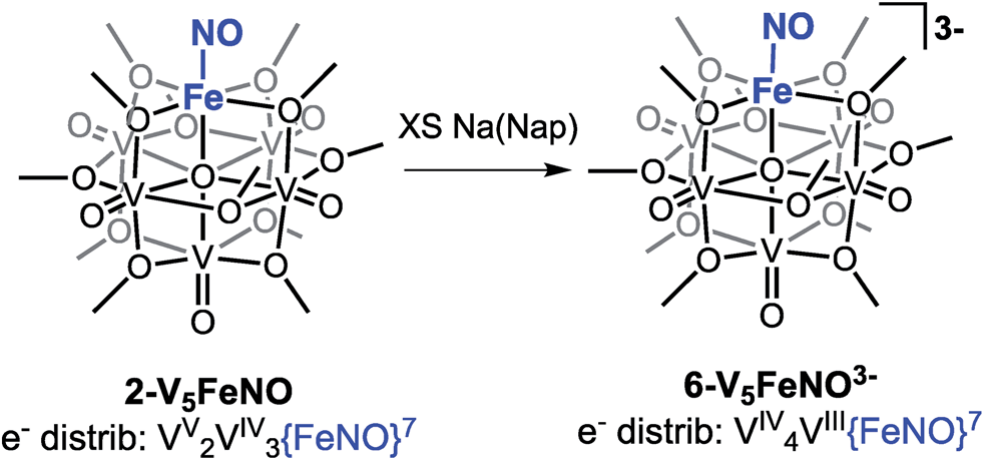
Attempted synthesis of complex **6-V_5_FeNO^3–^***via* the triple reduction of **2-V_5_FeNO**.

Reduction of **2-V_5_FeNO** in THF was attempted at low-temperatures with a freshly prepared solution of sodium naphthalenide. Stirring the solution for 1 hour results in apparent formation of a single product, as observed by ^1^H NMR spectroscopy (Fig. S7,† *δ* = 90.33, 34.48, 16.03 ppm in CD_3_CN). This three-resonance pattern in the product is similar to complexes **2–5**, suggesting clean formation of the desired fully-reduced {FeNO}POV–alkoxide (Scheme 3). However, multiple attempts to characterize the reduced cluster *via* Mössbauer spectroscopy resulted in identification of three distinct electronic environments for iron within the sample (Fig. S9, Table S1†). Despite the chemical reversibility observed for the most-reducing event in the CV of **2-V_5_FeNO**, and the seemingly straightforward ^1^H NMR spectrum of the product, the complicated Mössbauer spectrum suggests that substantial decomposition occurs upon attempts to chemically generate [V_5_O_6_(OCH_3_)_12_{FeNO}]^3–^ (**6-V_5_FeNO^3–^**), to paramagnetic, ^1^H NMR-silent byproducts.

**Fig. 6:**
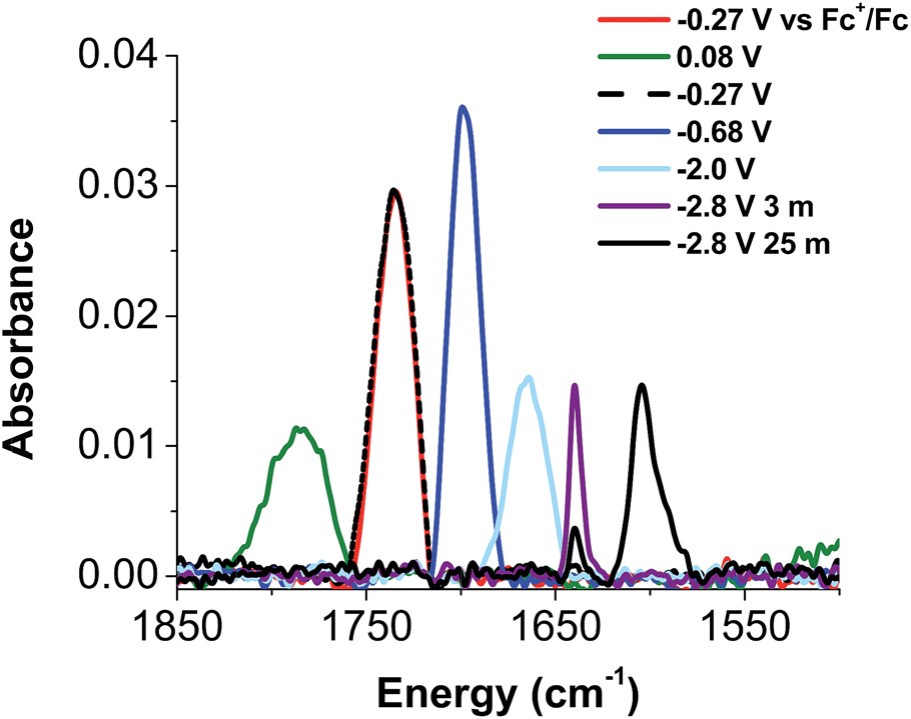
IR-SEC data of complexes **2-V_5_FeNO** measured at –0.27 V (**2-V_5_FeNO**, red), 0.08 V (**5-V_5_FeNO^+^**, green), –0.68 V (**3-V_5_FeNO^–^**, blue), –2.0 V (**4-V_5_FeNO^2–^**, light blue), –2.8 V at 3 min (**6-V_5_FeNO^3–^**, purple), –2.8 V at 25 min (black).

Given the apparent thermal instability of the fully reduced cluster, *in situ* electroanalytical techniques were employed for characterization of the {FeNO}POV–alkoxide clusters (Fig. 6, Table 2). Infrared-spectroelectrochemistry (IR-SEC) allows IR active modes in a compound of interest to be monitored with respect to changes in electrochemical potential as a function of time.^64^,^65^ To correlate the electrochemical response directly to the independently synthesized compounds, we started with the electrochemical generation of complexes **2–5**. Indeed, a 3 mM solution of **2-V_5_FeNO** in 0.4 M TBAPF_6_/THF supporting electrolyte at –0.27 V *vs.* Fc/Fc^+^ shows an IR absorbance at 1735 cm^–1^. This value compares well with the IR data from the independently synthesized sample (1732 cm^–1^, Fig. 3, Table 1). When the applied cell potential was brought to 0.08 V *vs.* Fc/Fc^+^, an absorption band at 1785 cm^–1^ appears and grows in intensity with the concomitant loss of the band at 1735 cm^–1^, consistent with the electrochemical generation of the one-electron oxidized species **5-V_5_FeNO^1+^** (*v*(NO) (ATR) = 1780 cm^–1^). Upon returning to –0.27 V, the *ν*(NO) of the starting material **2-V_5_FeNO** at 1735 cm^–1^ was regenerated with the loss of the band at 1785 cm^–1^ (Fig. 6), indicating good reversibility of this electrochemical process. At more negative potentials, –0.68 V *vs.* Fc/Fc^+^, an absorption at 1699 cm^–1^ appears with the disappearance of the band at 1735 cm^–1^, consistent with the electrochemical generation of the one-electron reduced species **3-V_5_FeNO^1–^** (*v*(NO) (ATR) = 1709 cm^–1^). At –2.0 V *vs.* Fc/Fc^+^, a new IR absorbance band at 1664 cm^–1^ is observed to grow in intensity with the loss of the band at 1699 cm^–1^, indicating formation of **4-V_5_FeNO^2–^** (*v*(NO) (ATR) = 1683 cm^–1^).

**Table 2:** Comparison for *v*(NO) values for chemically prepared {FeNO}POV–alkoxide clusters and values obtained from IR-SEC data

Complex	Chemical reduction/oxidation *v*(NO) (cm^–1^)	IR-SEC *v*(NO) (cm^–1^)
**5-V_5_FeNO^+^**	1780	1785
**2-V_5_FeNO**	1732	1735
**3-V_5_FeNO^–^**	1709	1699
**4-V_5_FeNO^2–^**	1683	1664
**6-V_5_FeNO^3–^**	—^*a*^	1640 (3 min) 1604 (25 min)^*b*^

^*a*^We were unable to isolate complex **6-V_5_FeNO^3–^** chemically, therefore the IR spectrum of this compound was not obtained.

^*b*^ *v*(NO) absorbance corresponds to an irreversible chemical transformation that occurs following formation of **6-V_5_FeNO^3–^**.

To probe the formation of the fully reduced cluster, [V_5_O_6_(OCH_3_)_12_FeNO]^3–^ (**6-V_5_FeNO^3–^**), *via* IR-SEC, the cell potential was held at –2.8 V *vs.* Fc/Fc^+^. An IR absorption band appears at 1640 cm^–1^ and increases in intensity with loss of the band at 1664 cm^–1^. After holding a potential of –2.8 V for 25 min, the band noted at 1640 cm^–1^ corresponding to tri-reduced complex, **6-V_5_FeNO^3–^**, was nearly consumed, with concomitant formation of a new complex with a *v*(NO) located at 1604 cm^–1^. This new species is indicative of a subsequent chemical reorganization of the {FeNO}POV–alkoxide cluster, as attempts to return the potential toward oxidizing regimes did not result in regeneration of **2-V_5_FeNO**.

The instability of **6-V_5_FeNO^3–^**, as confirmed by IR-SEC, is consistent with our experimental data. In the chemical reduction of **2-V_5_FeNO** with excess sodium naphthalenide, the reaction mixture is stirred for one hour, well beyond the lifetime of the reduced cluster as suggested by IR SEC (∼25 min). Attempts to chemically re-oxidize the product mixture isolated from chemical reduction were unsuccessful, indicating that over the course of an hour, complex **6-V_5_FeNO^3–^** disproportionates to new products with vastly different electronic properties.

The small change in *v*(NO) (∼25 cm^–1^) observed in the IR-SEC data of the transiently generated tri-reduced cluster (**6-V_5_FeNO^3–^**) suggests that despite the large Δ*E*_1/2_ of the most reducing event, the final reduction likely is localized to the POV–alkoxide metalloligand, as opposed to the {FeNO} subunit. An additional reduction across the POV–alkoxide scaffold requires formation of an FePOV–alkoxide cluster with a single V(iii) ion. Accessing a V(iii) ion within an iron-functionalized POV–alkoxide scaffold has been reported previously by our laboratory,^25^ however, the significant peak-to-peak separation observed in the CV of complex **2-V_5_FeNO** was not observed in the case of the parent FePOV–alkoxide cluster, **1-V_5_Fe**. In the case of the nitrosyl functionalized cluster, accessing the V(iii) ion within the POV–alkoxide scaffold only occurs at extremely reducing potentials, and immediately affords disproportionation. The seemingly straight-forward ^1^H NMR spectrum of the product of decomposition and the reduced *v*(NO) observed by IR-SEC suggests that degradation of **6-V_5_FeNO^3–^** results in formation of an FePOV–alkoxide cluster with a chemically activated nitrosyl unit. Current investigations into the molecular composition of this highly reactive by-product are underway.

## Conclusions

In summary, a series of NO-bound FePOV–alkoxide clusters, **5-V_5_FeNO^+^**, **2-V_5_FeNO**, **3-V_5_FeNO^–^**, and **4-V_5_FeNO^2–^**, varying in oxidation state by a single electron have been synthesized and systematically characterized using a variety of spectroscopic techniques. The charge distributions of complexes **2–5** were determined, revealing electron transfer from the POV–alkoxide scaffold to the iron–nitrosyl moiety upon coordination of NO. These results demonstrate that the metal-oxide metalloligand is capable of providing electron density to the iron centre for substrate coordination and activation.

The formation of {FeNO}^7^ motifs in FePOV–alkoxide clusters illustrates our initial foray into utilizing metal-oxide scaffolds as redox reservoirs for first-row transition metal complexes. The diffuse electron density of the POV–alkoxide scaffold, coupled with a suitable vacant site on the heterometal, enables coordination and the reduction of NO at a ferric centre. Future studies will expand upon this seminal work—extending it to other small molecule substrates and uncovering new approaches to mediating multielectron transformations using earth abundant elements.

## Experimental

### General considerations

All manipulations were carried out in the absence of water and oxygen in a UniLab MBraun inert atmosphere glovebox under a dinitrogen atmosphere. Glassware was oven dried for a minimum of 4 h and cooled in an evacuated antechamber prior to use. Unless otherwise noted, solvents were dried and deoxygenated on a Glass Contour System (Pure Process Technology, LLC) and stored over activated 3 Å molecular sieves (Fisher Scientific). Celite 545 (J. T. Baker) was dried in a Schlenk flask for at least 14 h at 150 °C under vacuum prior to use. Sodium (Na, 99%+), Silver perchlorate (AgClO_4_, anhydrous, 97%) and bis(cyclopentadienyl)cobalt(ii) (CoCp_2_, 98%) were purchased from Sigma-Aldrich and used as received. Nitric Oxide (NO, Matheson Gas Products, Inc. 99%) was purified according to literature precedent.^66^ [V_5_O_6_(OCH_3_)_12_Fe]^+^, [V_5_O_6_(OCH_3_)_12_Fe] (**1-V_5_Fe**), [V_5_O_6_(OCH_3_)_12_Fe]^–^, and [V_5_O_6_(OCH_3_)_12_Fe]^2–^ were synthesized according to our previous report.^24^,^25^

^1^H NMR spectra were recorded on a Bruker DPX-500 MHz spectrometer locked on the signal of deuterated solvents. All chemical shifts were reported relative to the peak of a residual ^1^H signal in deuterated solvents. CD_3_CN and CDCl_3_ were purchased from Cambridge Isotope Laboratories, degassed by three freeze–pump–thaw cycles, and stored over activated 3 Å molecular sieves. Infrared (FT-IR, ATR) spectra of complexes were recorded on a Shimadzu IR Affinity-1 Fourier Transform infrared spectrophotometer and are reported in wavenumbers (cm^–1^). Electronic absorption measurements were recorded at room temperature in anhydrous acetonitrile in a sealed 1 cm quartz cuvette with an Agilent Cary 60 UV-Vis spectrophotometer. Elemental analyses were performed on a PerkinElmer 2400 Series II Analyzer at the CENTC Elemental Analysis Facility (University of Rochester).

Cyclic Voltammetry experiments were recorded with a CH Instruments Inc. 410c time-resolved electrochemical quartz crystal microbalance. All measurements were performed in a three-electrode system cell configuration that consisted of a glassy-carbon (*ø* = 3.0 mm) working electrode, a Pt wire counter electrode, and an Ag/AgCl wire reference electrode. All electrochemical measurements were performed at room temperature in a N_2_-filled glovebox. A 0.4 M ^*n*^Bu_4_NPF_6_ solution (anhydrous THF) was used as the electrolyte solution. All redox events were referenced against the ferrocene/ferrocenium (Fc/Fc^+^) redox couple.

All samples for ^57^Fe Mössbauer spectroscopy were run as isolated solid samples made from natural abundance iron. All samples were prepared in an inert-atmosphere glovebox equipped with a liquid-nitrogen fill port. This enables freezing of the samples to 77 K within the glovebox. Samples were loaded into a Delrin Mössbauer cup for measurements and loaded under liquid nitrogen. ^57^Fe Mössbauer measurements were performed using a SEE Co. MS4 Mössbauer spectrometer integrated with a Janis SVT-400T He/N_2_ cryostat for measurements at 80 K. Isomer shifts were determined relative to α-iron at 298 K. All Mössbauer spectra were fit using the program WMoss (SEE Co.).

Samples for Electron Paramagnetic Resonance analysis were prepared in an inert atmosphere glovebox (N_2_). EPR samples were prepared as solutions in acetonitrile and loaded into 4 mm OD Suprasil quartz EPR tubes from Wilmad Labglass. X-band EPR spectra were collected at 10 K on a Bruker EMXplus spectrometer equipped with a 4119HS cavity and an Oxford ESR-900 helium flow cryostat. Instrumental parameters employed for all samples were as follows: 1 mW power; 80 ms time constant; 8 G modulation amplitude; 9.38 GHz frequency; and 100 kHz modulation frequency.

IR Spectroelectrochemistry (IR-SEC) experiments were conducted using a custom cell based on a previously published design.^67^–^69^ The three-electrode set-up consists of an inner glassy carbon working electrode disc (10 mm diameter), a central circular silver bare metal pseudoreference electrode, and an outer circular glassy carbon counter electrode embedded within a PEEK block. All data were referenced to an internal ferrocene standard (ferricenium/ferrocene reduction potential under stated conditions; obtained by taking a CV with the cell prior to injecting analyte for IR-SEC experiments) unless otherwise specified. IR-SEC solutions were prepared inside a glovebox under N_2_ atmosphere and injected in IR-SEC cell using an SGE airtight syringe. IR spectra were collected using a Bruker Vertex 80 spectrometer with a liquid nitrogen-cooled detector.

### Synthesis of {(VO)_5_O(OCH_3_)_12_FeNO} (**2-V_5_FeNO**)

In the glovebox, a 250 mL Schlenk flask was charged with **1-V_5_Fe** (0.119 g, 0.152 mmol) and 40 mL CH_2_Cl_2_. The nitrogen atmosphere was removed with two freeze–pump–thaw cycles, and one atmosphere of purified NO gas (normal temperature and pressure) was added *via* to the evacuated flask. The solution was vigorously stirred for three days, resulting in a colour change from dark-green to brown. Volatiles were removed under reduced pressure, and the flask was taken back into the glovebox. The product was extracted with diethyl ether (Et_2_O, 10 mL × 3), followed by extraction with *n*-pentane (10 mL × 3). Removal of solvent under reduced pressure results in isolation of the product, **2-V_5_FeNO** (0.074 g, 0.091 mmol, 60%). ^1^H NMR (500 MHz, CD_3_CN): *δ* = 127.44 (s, *J* = 283 Hz), 20.12 (s, *J* = 84 Hz), 13.86 (s, *J* = 94 Hz). FT-IR (ATR, cm^–1^): 2920 (C–H), 2814 (C–H), 1730 (N=O), 1022 (O–CH_3_), 968 (V=O). UV-Vis [CH_3_CN; *λ*, nm (*ε*, M^–1^ cm^–1^)]: 382 (5.06 × 10^3^), 992 (5.80 × 10^2^). Elemental analysis for C_12_H_36_NO_19_V_5_Fe (MW = 808.96 g mol^–1^) calcd (%): C, 17.82; H, 4.49; N, 1.73. Found (%): C, 18.16; H, 4.13; N, 1.36.

### Synthesis of CoCp_2_{(VO)_5_O(OCH_3_)_12_FeNO} (**3-V_5_FeNO^–^**)

#### Method A

A 20 mL scintillation vial was charged with **2-V_5_FeNO** (0.164 g, 0.203 mmol) and 16 mL THF. In a separate vial, cobaltocene (CoCp_2_, 0.037 g, 0.196 mmol) was dissolved in 4 mL THF and added dropwise to the cluster solution with vigorous stirring. No obvious colour change was noticed, and the solution was stirred vigorously for additional 2 h. The solution was filtered over a bed of celite (1 cm), and the solvent was removed under reduced pressure. The solid residue was washed with toluene (5 mL × 3) and pentane (5 mL × 3). The brown solid was then dried under vacuum to yield complex **3-V_5_FeNO^–^** in excellent yield (0.182 g, 0.182 mmol, 90%). ^1^H NMR (500 MHz, CD_3_CN): *δ* = 136.65 (s, *J* = 407 Hz), 20.28 (s, *J* = 171 Hz), 5.67 (s, *J* = 46 Hz). FT-IR (ATR, cm^–1^): 3082 (C–H), 2914 (C–H), 2806 (C–H), 1709 (N=O), 1032 (O–CH_3_), 951 (V=O). UV-Vis [CH_3_CN; *λ*, nm (*ε*, M^–1^ cm^–1^)]: 384 (2.68 × 10^3^), 992 (4.14 × 10^2^). Elemental analysis for C_22_H_46_NO_19_V_5_FeCo (MW = 998.08 g mol^–1^) calcd (%): C, 26.47; H, 4.65; N, 1.40. Found (%): C, 26.87; H, 4.43; N, 1.11.

#### Method B

In the glovebox, a 250 mL round bottom Schlenk flask was charged with K(VO)_5_O(OCH_3_)_12_Fe (0.130 g, 0.159 mmol) and 40 mL CH_2_Cl_2_/THF (1 : 1) mixture. The flask was capped with a septum, carefully taped, and removed from the glovebox. Purified NO gas (*ca.* 74 mL at normal temperature and pressure conditions) was added *via* gas-tight syringe. The solution was vigorously stirred for four days and afforded a colour change from dark-green to brown. The solvent was removed under reduced pressure, and the flask was taken back into the glovebox. Copious amount of CH_2_Cl_2_ was added to extract the solid residue until the extraction turned nearly colourless, followed by removal of the volatiles to yield **3-V_5_FeNO^–^** as a brown powder (0.050 g, 0.059 mmol, 37%). ^1^H NMR and FT-IR spectra collected for the product are identical to that observed for method A.

### Synthesis of (CoCp_2_)_2_{(VO)_5_O(OCH_3_)_12_FeNO} (**4-V_5_FeNO^2–^**)

A 20 mL scintillation vial was charged with **2-V_5_FeNO** (0.124 g, 0.153 mmol) and 16 mL THF. In a separate vial, cobaltocene (CoCp_2_, 59 mg, 0.311 mmol) was dissolved in 4 mL THF and added dropwise to the cluster solution while stirring. A dark grey-brown precipitate started to form within 5 minutes. The mixture was stirred vigorously for an additional 2 h. The precipitate was collected and washed with THF until the filtrate was nearly colourless. The grey-brown solid was dried under vacuum to yield complex **4-V_5_FeNO^2–^** in good yield (0.148 g, 0.125 mmol, 81%). ^1^H NMR (500 MHz, CD_3_CN): *δ* = 135.68 (s, *J* = 436 Hz), 23.54 (s, *J* = 142 Hz), 22.29 (s, *J* = 128 Hz), 5.68 (s, *J* = 38 Hz). FT-IR (ATR, cm^–1^): 3078 (C–H), 2909 (C–H), 2803 (C–H), 1683 (N=O), 1055 (O–CH_3_), 935 (V=O). UV-Vis [CH_3_CN; *λ*, nm (*ε*, M^–1^ cm^–1^)]: 435 (1.78 × 10^3^), 542 (6.61 × 10^2^). Elemental analysis for C_32_H_56_NO_19_V_5_Fe_2_ (MW = 1187.20 g mol^–1^) calcd (%): C, 32.37; H, 4.75; N, 1.18. Found (%): C, 32.47; H, 4.51; N, 1.22.

### Synthesis of {(VO)_5_O(OCH_3_)_12_FeNO}ClO_4_ (**5-V_5_FeNO^+^**)

#### Method A

A 20 mL scintillation vial was charged with **2-V_5_FeNO** (0.042 g, 0.052 mmol) and 6 mL THF. The vial was placed in a cold well cooled to –40 °C. In a separate vial, silver perchlorate, (AgClO_4_, 0.011 g, 0.053 mmol) was dissolved in 2 mL THF, placed in the cold well for 10 minutes, and added dropwise to the cold cluster solution with vigorous stirring. The mixture was warmed to room temperature, during which time a dark-grey precipitate started to form. The reaction was subsequently stirred for 2 h to ensure completion. The solution was filtered over celite (1 cm), and the solvent was removed under reduced pressure, followed by extraction of the solid residue with diethyl ether. Volatiles were removed under vacuum to yield **5-V_5_FeNO^+^** as a brown powder in good yield (0.037 g, 0.041 mmol, 78%). ^1^H NMR (500 MHz, CDCl_3_): *δ* = 112.11 (s, *J* = 352 Hz), 13.33 (s, *J* = 102 Hz), 12.01 (s, *J* = 112 Hz). FT-IR (ATR, cm^–1^): 2926 (C–H), 2820 (C–H), 1780 (N=O), 1260 (Cl=O), 1003 (O–CH_3_), 975 (V=O). UV-Vis [CH_3_CN; *λ*, nm (*ε*, M^–1^ cm^–1^)]: 386 (1.08 × 10^4^), 992 (6.68 × 10^2^). Elemental analysis of complex **5-V_5_FeNO^+^** was not obtained because the compound is not sufficiently stable at room temperature.

#### Method B

In the glovebox, a 250 mL round bottom Schlenk flask was charged with (VO)_5_O(OCH_3_)_12_FeClO_4_ (0.132 g, 0.150 mmol) and 40 mL CH_2_Cl_2_. The flask was capped with a septum, carefully taped, and removed from the glovebox. Purified NO gas (*ca.* 74 mL at normal temperature and pressure conditions) was added *via* gas-tight syringe. The solution was vigorously stirred overnight and afforded a colour change from dark green to dark brown. The solvent was removed under reduced pressure, and the flask was taken back into the glovebox. Toluene (10 mL × 3) was added to extract the solid residue, followed by removal of the volatiles to yield **5-V_5_FeNOClO_4_** as a brown powder (0.088 g, 0.109 mmol, 71%). ^1^H NMR and FT-IR spectra collected for the product are identical to that observed for method A.

### Chemical reduction of **2-V_5_FeNO** with Na(Nap)

A 20 mL Scintillation vial was charged with 0.028 g naphthalene (0.218 mmol) and 6 mL THF, to which pieces of freshly cut sodium metal (0.073 g, 3.167 mmol) were added. The mixture was stirred at room temperature for 3 hours, affording a dark green solution of sodium naphthanelide. This solution was decanted into a fresh 20 mL Scintillation vial and frozen in an 80 K cold well. In a separate vial, **2-V_5_FeNO** (0.054 g, 0.067 mmol) was dissolved in 2 mL of THF, and the solution was frozen at 80 K. The **2-V_5_FeNO** solution was allowed to thaw and was added to the frozen sodium naphthanelide solution along with an additional 2 mL thawed THF to complete the transfer. The reaction was stirred vigorously and allowed to warm to room temperature for 1 hour. The resulting brown solution was filtered over a bed of celite (0.5 cm), the volatiles were removed, and the brown solid was washed with toluene (5 mL × 2).

## Conflicts of interest

There are no conflicts of interest to declare.

## Supplementary Material

Supplementary information

## References

[cit1] Zweier J. L., Li H., Samouilov A., Liu X. (2010). Nitric Oxide.

[cit2] Wasser I. M., de Vries S., Moënne-Loccoz P., Schröder I., Karlin K. D. (2002). Chem. Rev..

[cit3] Møller J. K. S., Skibsted L. H. (2002). Chem. Rev..

[cit4] Richter-Addo G. B. and Legzdins P., Metal Nitrosyls, Oxford University Press, New York, NY, 1992.

[cit5] Galloway J. N., Dentener F. J., Capone D. G., Boyer E. W., Howarth R. W., Seitzinger S. P., Asner G. P., Cleveland C. C., Green P. A., Holland E. A., Karl D. M., Michaels A. F., Porter J. H., Townsend A. R., Vöosmarty C. J. (2004). Biogeochem..

[cit6] Kurts J. D. M. (2007). Dalton Trans..

[cit7] Watmough N. J., Field S. J., Hughes R. J. L., Richardson D. J. (2009). Biochem. Soc. Trans..

[cit8] Sato N., Ishii S., Sugimoto H., Hino T., Fukumori Y., Sako Y., Shiro Y., Tosha T. (2013). Proteins: Struct., Funct., Bioinf..

[cit9] Moenne-Loccoz P. (2007). Nat. Prod. Rep..

[cit10] Timmons A. J., Symes M. D. (2015). Chem. Soc. Rev..

[cit11] Berto T. C., Speelman A. L., Zheng S., Lehnert N. (2013). Coord. Chem. Rev..

[cit12] Chakraborty S., Reed J., Sage J. T., Branagan N. C., Petrik I. D., Miner K. D., Hu M. Y., Zhao J., Alp E. E., Lu Y. (2015). Inorg. Chem..

[cit13] Silaghi-Dumitrescu R., Kurtz D. M., Ljungdahl L. G., Lanzilotta W. N. (2005). Biochem..

[cit14] Ferreira K. N., Iverson T. M., Maghlaoui K., Barber J., Iwata S. (2004). Science.

[cit15] Einsle O., Tezcan F. A., Andrade S. L. A., Schmid B., Yoshida M., Howard J. B., Rees D. C. (2002). Science.

[cit16] Schilter D., Camara J. M., Huynh M. T., Hammes-Schiffer S., Rauchfuss T. B. (2016). Chem. Rev..

[cit17] McGlynn S. E., Mulder D. W., Shepard E. M., Broderick J. B., Peters J. W. (2009). Dalton Trans..

[cit18] Dobbek H., Svetlitchnyi V., Gremer L., Huber R., Meyer O. (2001). Science.

[cit19] Beinert H., Holm R. H., Münck E. (1997). Science.

[cit20] Brandt U. (2006). Annu. Rev. Biochem..

[cit21] Wang Y.-G., Yoon Y., Glezakou V.-A., Li J., Rousseau R. (2013). J. Am. Chem. Soc..

[cit22] Rodriguez J. A., Liu P., Stacchiola D. J., Senanayake S. D., White M. G., Chen J. G. (2015). ACS Catal..

[cit23] Ruiz Puigdollers A., Schlexer P., Tosoni S., Pacchioni G. (2017). ACS Catal..

[cit24] Li F., VanGelder L. E., Brennessel W. W., Matson E. M. (2016). Inorg. Chem..

[cit25] Li F., Carpenter S. H., Higgins R. F., Hitt M. G., Brennessel W. W., Ferrier M. G., Cary S. K., Lezama-Pacheco J. S., Wright J. T., Stein B. W., Shores M. P., Neidig M. L., Kozimor S. A., Matson E. M. (2017). Inorg. Chem..

[cit26] Kastner K., Margraf J. T., Clark T., Streb C. (2014). Chem.–Eur. J..

[cit27] Powers T. M., Gu N. X., Fout A. R., Baldwin A. M., Hernández Sánchez R., Alfonso D. M., Chen Y.-S., Zheng S.-L., Betley T. A. (2013). J. Am. Chem. Soc..

[cit28] Eames E. V., Hernández Sánchez R., Betley T. A. (2013). Inorg. Chem..

[cit29] Kanady J. S., Tsui E. Y., Day M. W., Agapie T. (2011). Science.

[cit30] Tsui E. Y., Tran R., Yano J., Agapie T. (2013). Nat. Chem..

[cit31] Herbert D. E., Lionetti D., Rittle J., Agapie T. (2013). J. Am. Chem. Soc..

[cit32] Speelman A. L., Zhang B., Krebs C., Lehnert N. (2016). Angew. Chem., Int. Ed..

[cit33] Hayton T. W., Legzdins P., Sharp W. B. (2002). Chem. Rev..

[cit34] Pierson R. H., Fletcher A. N., Gantz E. S. C. (1956). Anal. Chem..

[cit35] Spagnolo V., Kosterev A. A., Dong L., Lewicki R., Tittel F. K. (2010). Appl. Phys. B.

[cit36] Speelman A. L., Lehnert N. (2013). Angew. Chem., Int. Ed..

[cit37] Serres R. G., Grapperhaus C. A., Bothe E., Bill E., Weyhermüller T., Neese F., Wieghardt K. (2004). J. Am. Chem. Soc..

[cit38] Hauser C., Glaser T., Bill E., Weyhermüller T., Wieghardt K. (2000). J. Am. Chem. Soc..

[cit39] Victor E., Lippard S. J. (2014). Inorg. Chem..

[cit40] Harrop T. C., Tonzetich Z. J., Reisner E., Lippard S. J. (2008). J. Am. Chem. Soc..

[cit41] Daniel C., Hartl H. (2009). J. Am. Chem. Soc..

[cit42] Daniel C., Hartl H. (2005). J. Am. Chem. Soc..

[cit43] Scheidt W. R., Ellison M. K. (1999). Acc. Chem. Res..

[cit44] Cooper C. E. (1999). Biochim. Biophys. Acta.

[cit45] Salomão G. C., Olsen M. H. N., Drago V., Fernandes C., Cardozo Filho L., Antunes O. A. C. (2007). Catal. Commun..

[cit46] Spandl J., Daniel C., Brüdgam I., Hartl H. (2003). Angew. Chem., Int. Ed..

[cit47] Hernández Sánchez R., Zheng S.-L., Betley T. A. (2015). J. Am. Chem. Soc..

[cit48] Gagne R. R., Spiro C. L. (1980). J. Am. Chem. Soc..

[cit49] Hu B., Li J. (2015). Angew. Chem., Int. Ed..

[cit50] McCleverty J. A., Atherton N. M., Locke J., Wharton E. J., Winscom C. J. (1967). J. Am. Chem. Soc..

[cit51] Ghosh P., Stobie K., Bill E., Bothe E., Weyhermüller T., Ward M. D., McCleverty J. A., Wieghardt K. (2007). Inorg. Chem..

[cit52] Robin M. B. and Day P., in Adv. Inorg. Chem. and Radiochem., ed. H. J. Emeléus and A. G. Sharpe, Academic Press, 1968, vol. 10, pp. 247–422.

[cit53] Enemark J. H., Feltham R. D. (1974). Coord. Chem. Rev..

[cit54] Augustyniak-Jabłokow M. A., Daniel C., Hartl H., Spandl J., Yablokov Y. V. (2008). Inorg. Chem..

[cit55] Arnett C. H., Chalkley M. J., Agapie T. (2018). J. Am. Chem. Soc..

[cit56] de Ruiter G., Thompson N. B., Lionetti D., Agapie T. (2015). J. Am. Chem. Soc..

[cit57] Reed C. J., Agapie T. (2017). Inorg. Chem..

[cit58] Toth J. E., Anson F. C. (1989). J. Am. Chem. Soc..

[cit59] Pratt H. D., Pratt W. R., Fang X., Hudak N. S., Anderson T. M. (2014). Electrochim. Acta.

[cit60] Berto T. C., Hoffman M. B., Murata Y., Landenberger K. B., Alp E. E., Zhao J., Lehnert N. (2011). J. Am. Chem. Soc..

[cit61] Kupper C., Rees J. A., Dechert S., DeBeer S., Meyer F. (2016). J. Am. Chem. Soc..

[cit62] Confer A. M., McQuilken A. C., Matsumura H., Moënne-Loccoz P., Goldberg D. P. (2017). J. Am. Chem. Soc..

[cit63] Montenegro A. C., Amorebieta V. T., Slep L. D., Martín D. F., Roncaroli F., Murgida D. H., Bari S. E., Olabe J. A. (2009). Angew. Chem., Int. Ed..

[cit64] Ashley K., Pons S. (1988). Chem. Rev..

[cit65] Kaim W., Fiedler J. (2009). Chem. Soc. Rev..

[cit66] Speelman A. L., Zhang B., Silakov A., Skodje K. M., Alp E. E., Zhao J., Hu M. Y., Kim E., Krebs C., Lehnert N. (2016). Inorg. Chem..

[cit67] Zavarine I. S., Kubiak C. P. (2001). J. Electroanal. Chem..

[cit68] Machan C. W., Sampson M. D., Chabolla S. A., Dang T., Kubiak C. P. (2014). Organometallics.

[cit69] Nichols A. W., Chatterjee S., Sabat M., Machan C. W. (2018). Inorg. Chem..

