# Supplementary material for: Nitric oxide activation facilitated by cooperative multimetallic electron transfer within an iron-functionalized polyoxovanadate–alkoxide cluster
Source: Chem Sci. 2018 Jul 2;9(30):6379–89. doi: 10.1039/c8sc00987b (PMC6115649; doi:10.1039/c8sc00987b)
Supplement: Supplementary file 1 [file SC-009-C8SC00987B-s001.pdf]

Electronic Supplementary Material (ESI) for Chemical Science.
This journal is © The Royal Society of Chemistry 2018

Nitric Oxide activation facilitated by cooperative multimetallic electron transfer within an iron-functionalized polyoxovanadate-alkoxide cluster

Feng Li,[a] Rachel L. Meyer,[a] Stephanie H. Carpenter,[a] Lauren E. VanGelder,[a] Aza W. Nichols,[b] Charles W. Machan,[b] Michael L. Neidig,[a] and Ellen M. Matson[a]*

[a] Department of Chemistry, University of Rochester, Rochester, New York 14627, United States
[b] Department of Chemistry, University of Virginia, Charlottesville, Virginia 22904, United States

Supporting Information Table of Contents:

Figure S1. ^{1}H NMR Spectrum of **2-V$_5$FeNO.** S2
Figure S2. Electrochemical data to test the reversibility of redox couples observed for complex **2-V$_5$FeNO.** S2
Figure S3. ^{1}H NMR Spectrum of **3-CoCp$_2$V$_5$FeNO.** S3
Figure S4. ^{1}H NMR Spectrum of **4-(CoCp$_2$)$_2$V$_5$FeNO.** S3
Figure S5. ^{1}H NMR Spectrum of **5-V$_5$FeNOClO$_4$.** S4
Figure S6. EPR Spectrum of **3-(CoCp)$_2$V$_5$FeNO**. S4
Figure S7. ^{1}H NMR Spectrum of **2-V$_5$FeNO** chemically reduced with Na(Napth) S5
Figure S8. Electronic Absorption Spectrum of **6-Na$_3$V$_5$FeNO.** S5
Figure S9. ^{57}Fe Mössbauer Spectrum of **2-V$_5$FeNO** chemically reduced with Na(Napth) S6
Table S1. ^{57}Fe Mössbauer parameters of product mixture of **2-V$_5$FeNO** chemically reduced with Na(Napth) S6

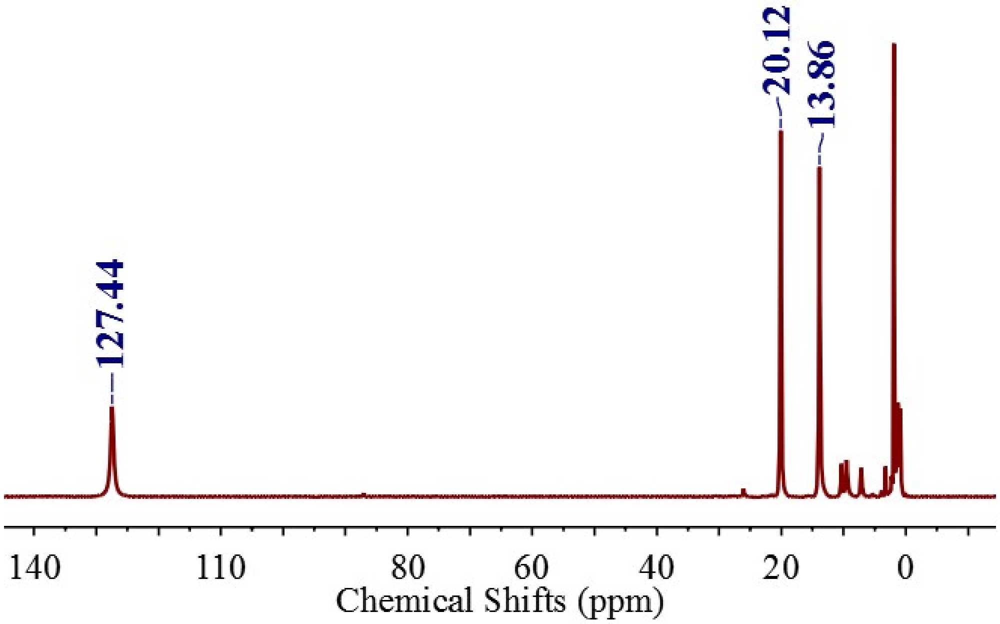

Figure S1. ^{1}H NMR (500 MHz) spectrum of **2-V_5FeNO** (CD_3CN, 21 °C).

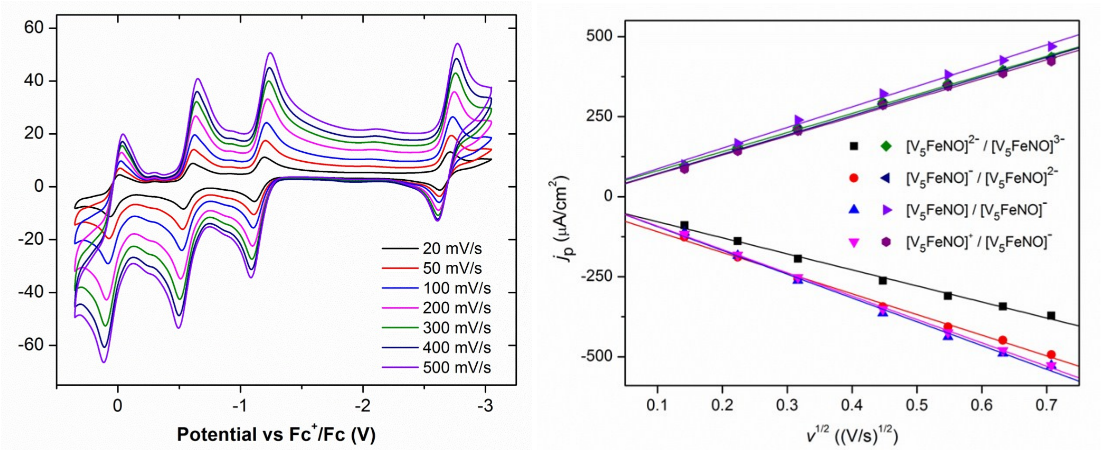

Figure S2. Electrochemical data to test the reversibility of redox couples observed for complex **2-V_5FeNO**. a.) Cyclic voltammetry of **2-V_5FeNO** (2 mM) taken at scan rates ranging from 20 to 500 mV/s in THF with a 0.4 M $[Bu_4N][PF_6]$ as the supporting electrolyte. b.) Plot of current density (j_p) vs the square root of the scan rate ($v^{1/2}$) for complex **2-V_5FeNO.**

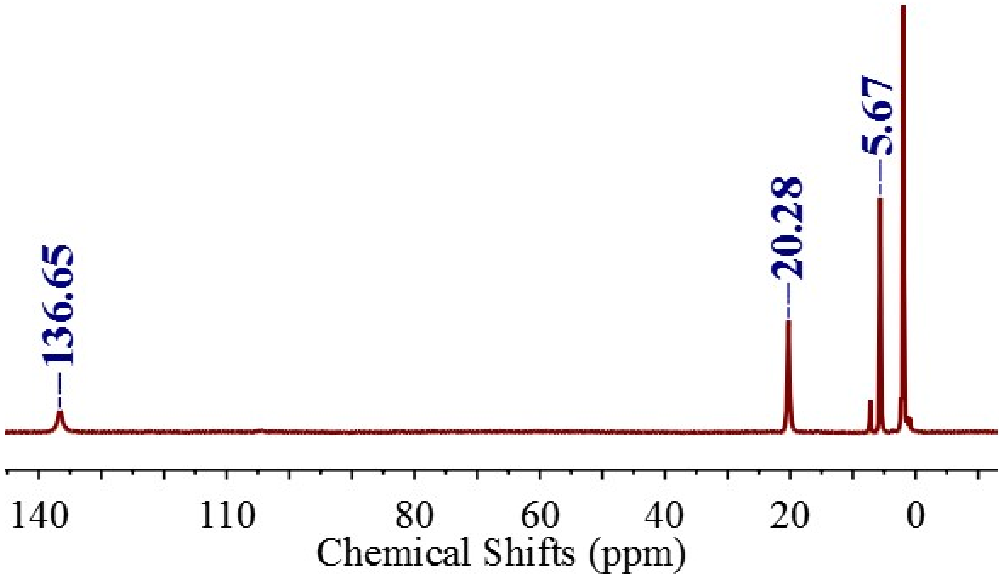

Figure S3. ^{1}H NMR (500 MHz) spectrum of **3-$CoCp_2V_5FeNO$** (CD_3CN, 21 °C).

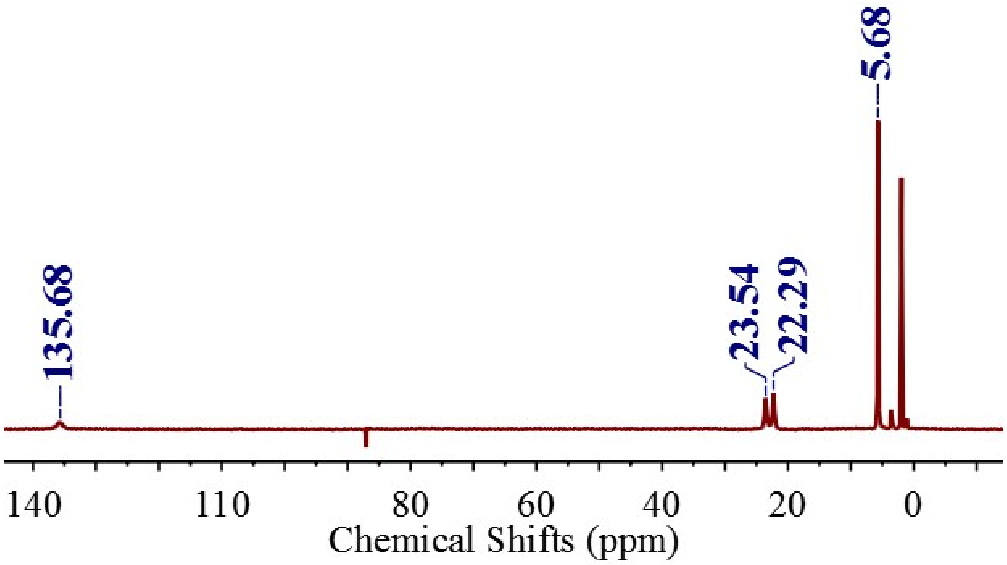

Figure S4. ^{1}H NMR (500 MHz) spectrum of **4-$(CoCp_2)_2V_5FeNO$** (CD_3CN, 21 °C).

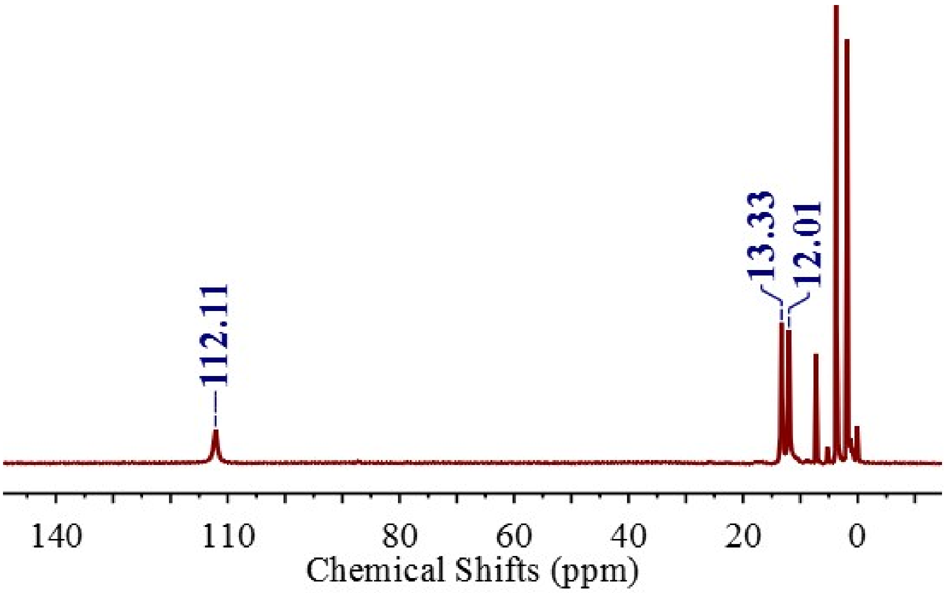

Figure S5. ^{1}H NMR (500 MHz) spectrum of **5-V$_5$FeNOClO$_4$** ($CDCl_3$, 21 °C).

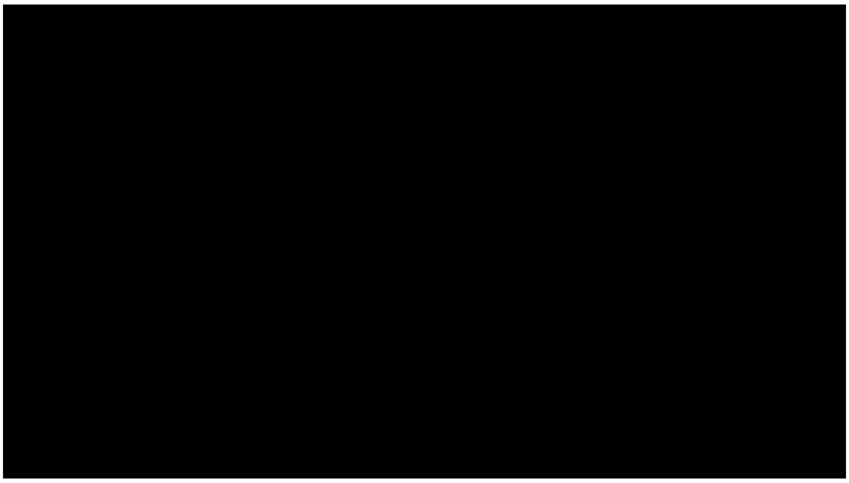

Figure S6. 10 K EPR spectrum of complex **3-V$_5$FeNO$^-$** measured in acetonitrile.

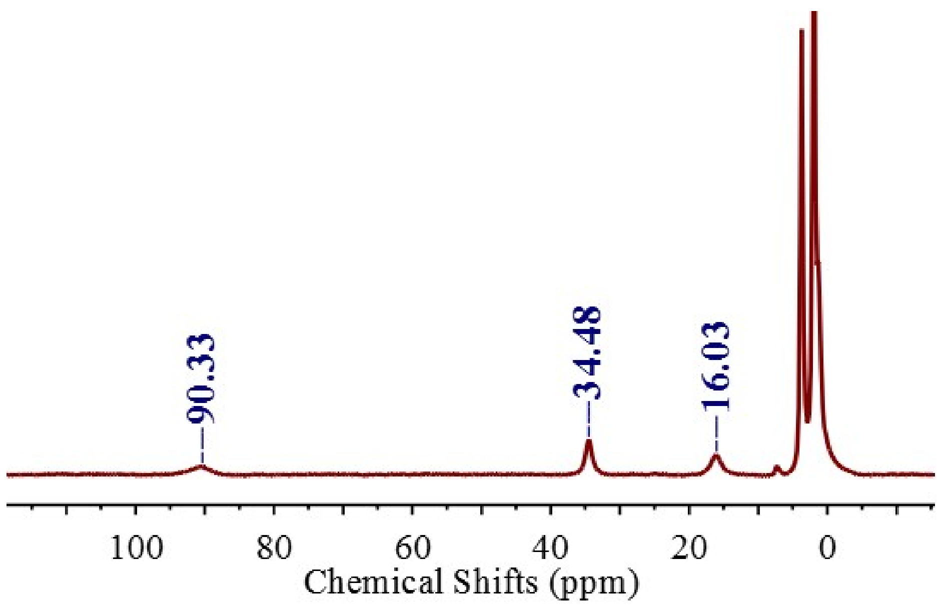

Figure S7. ^{1}H NMR (500 MHz) spectrum of **6- Na_3V_5FeNO** (CD_3CN, 21 °C).

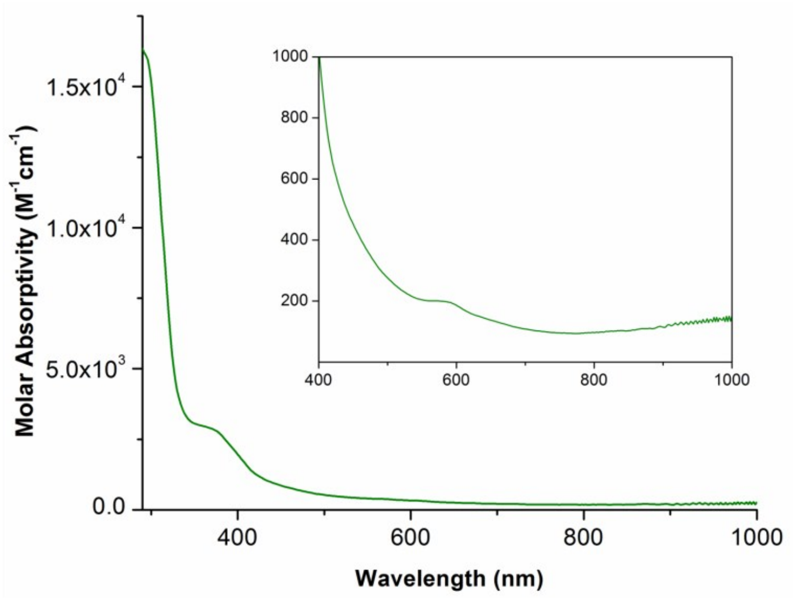

Figure S8. Electronic absorption spectrum of **6-Na_3V_5FeNO** collected in CH_3CN at 21 °C (the inset shows the low-energy region of the spectrum).

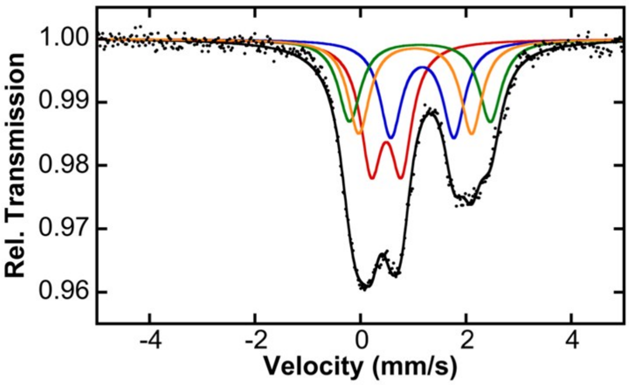

Figure S9. 80 K Mössbauer spectrum of the reduction of complex **2-V_5FeNO** with sodium napthalanide. Spectrum reveals decomposition of the fully reduced cluster.

Table S1. ^{57}Fe Mössbauer spectroscopic parameters of the decomposition of the product of the reduction of **2-V_5FeNO** with sodium napthalanide.

**Compound**	**Percentage of Sample**	**δ (mm s^{-1})**	**ΔE_Q (mm s^{-1})**
Blue Trace	24%	1.17	1.20
Green Trace	22%	1.13	2.67
Orange Trace	24%	1.04	2.14
Red Trace (**[$V_5O_6(OCH_3)_{12}$Fe]**$^-$) *Inorg. Chem.* **2017**, *56*, 7065-7080	30%	0.49	0.57
